# Menstrual Cycle Informed Psychotherapy: Preliminary Findings in Women with Spider Phobia

**DOI:** 10.1159/pps/aemag012

**Published:** 2026-09-05

**Authors:** Gianna Zorzini, Patricia Patra Ritter, Gianandrea Pallich, Ursula Galli, Ulrike Ehlert, Susanne Fischer

**Affiliations:** a Institute of Psychology, Clinical Psychology and Psychotherapy, University of Zurich, Zürich, Switzerland; b Reproductive Mental Health Research Unit, University of Regina, Regina, SK, Canada; c Department of Adult Psychiatry and Psychotherapy, University Hospital of Psychiatry Zurich, Zürich, Switzerland; d School of Medicine, Planetary Health, University of St. Gallen, St. Gallen, Switzerland

**Keywords:** Exposure therapy, Menstrual cycle, Progesterone, Estradiol, Phobia, Ovarian hormones

## Abstract

**Introduction:**

Anxiety disorders are the most frequent mental disorders worldwide. Although exposure therapy is the gold-standard treatment for anxiety disorders, almost half of patients fail to achieve sufficient improvements. While much has been learned about for whom psychotherapy works and why, far less is understood about when it works. Emerging evidence indicates that estradiol and progesterone may affect fear extinction, yet studies examining the effects of menstrual cycle phase on exposure therapy outcomes remain lacking. This study was the first to investigate to what extent biologically informed menstrual cycle phase affects exposure therapy outcomes in women with spider phobia.

**Method:**

N = 45 women with spider phobia in (1) the early to mid-follicular phase, (2) the mid-luteal phase, or (3) using hormonal contraceptives (HC), participated in a group-based exposure therapy. Symptoms were assessed at baseline, 1-week post-therapy, and at 12–14 weeks follow-up, using the Spider Phobia Questionnaire (SPQ), the Fear of Spiders Questionnaire (FSQ), and a behavioral approach test (BAT).

**Results:**

Both phobic fear and avoidance behavior improved significantly over time. Although improvement did not differ between menstrual cycle groups, higher progesterone levels on the day of exposure therapy were associated with less improvement in behavioral avoidance.

**Conclusion:**

The present findings provide preliminary evidence that ovarian hormones on the day of exposure therapy may affect exposure-based outcomes, particularly reductions in behavioral avoidance. More research in larger samples is needed to further investigate menstrual cycle phase effects in exposure-based treatment. If replicated, the findings would attest to the potential of bio-rhythm informed psychotherapy.

## Plain Language Summary

Anxiety disorders are the most common mental health conditions worldwide. One effective treatment is exposure therapy, in which people gradually and repeatedly confront their fears. For instance, someone afraid of spiders might work through a series of steps, from looking at pictures to eventually touching a spider. However, not everyone benefits from this therapy to the same extent, and the underlying reasons remain unclear. In people with a menstrual cycle, the hormones progesterone and estradiol rise and fall in a predictable monthly pattern, and animal studies suggest that hormones may affect how a person learns during therapy. This study investigated, for the first time, whether menstrual cycle phase and hormone levels on the day of exposure therapy are associated with how much people with a menstrual cycle benefit from it. Participants with a fear of spiders received exposure therapy during a cycle phase when estradiol and progesterone levels were low, when they were both high, or while using hormonal contraceptives. Their fear of spiders and their willingness to approach a spider were measured before therapy, 1 week later, and 3 months later. On average, participants showed clear and lasting improvements in both fear and avoidance, with no difference between the cycle groups. However, those with higher progesterone levels on the day of exposure therapy showed less improvement in avoidance. As the study was small, larger studies are needed to confirm these findings. Nevertheless, they provide initial evidence that a person's hormonal state may shape therapy outcomes.

## Introduction

Anxiety disorders are the most prevalent mental health conditions worldwide, with women showing approximately twice the lifetime risk compared to men [1–3]. Such gender differences are observed across anxiety disorders, including specific phobias such as spider phobia [4, 5]. Women also tend to experience a greater illness burden and higher rates of comorbidity [6], further underscoring the need for effective treatment options that address specific needs and complexities.

Exposure therapy is considered the gold-standard psychological treatment for anxiety and related disorders [7, 8]. It involves systematic confrontation with feared stimuli while preventing avoidance behavior [9], thereby promoting extinction learning and cognitive reappraisal of the feared stimulus [10]. However, treatment responses can vary substantially. While significant reductions in anxiety symptoms with large effect sizes have been observed [11], almost half of patients demonstrate insufficient therapeutic improvement [12]. Therefore, increasing attention has been directed toward identifying predictors of treatment response [13].

Emerging evidence indicates that biological factors may be promising determinants of therapeutic success [14]. Extinction learning relies on coordinated interactions between the amygdala, ventromedial prefrontal cortex (vmPFC), and hippocampus, which support fear inhibition and the consolidation and retrieval of safety memories [15]. While the amygdala is central for threat detection and fear expression [16], the vmPFC supports inhibitory control and extinction memory expression [17], and the hippocampus contributes to contextual modulation and memory retrieval [18]. Converging evidence suggests that ovarian hormones, particularly estradiol and progesterone, may modulate activation within this circuitry, ultimately affecting extinction learning. Merz et al. [19] demonstrated that sex hormones modulate the neural correlates of fear extinction, such that women using oral contraceptives (low endogenous estradiol and progesterone) showed altered neuronal responses during extinction, including in the amygdala and vmPFC, compared to naturally cycling women in the luteal phase (high endogenous estradiol and progesterone) and males. Ovarian hormones during exposure-based learning may, therefore, affect extinction-relevant neural processing, potentially translating into clinically meaningful differences in exposure-based interventions [20].

A meta-analysis [21] synthesizing findings from seven experimental studies examining the effects of ovarian hormones on extinction learning reported a significant positive association between endogenous estradiol and extinction recall. Specifically, five studies [22–26] reported that higher estradiol levels enhanced the retrieval of safety memories formed during extinction. However, only two of these studies accounted for menstrual cycle phase (e.g., early follicular phase, midcycle, and mid-luteal phase), which limits conclusions about cycle phase effects [22, 25]. Given the widespread distribution of estrogen receptors in the neuronal fear-circuit, estradiol on the day of extinction learning may, after binding to these receptors, affect exposure therapy outcomes by mediating the underlying molecular activity [27]. This is supported by neurobiological findings that estradiol is associated with increased activation of the vmPFC and related fear extinction circuitry during extinction learning and recall, ultimately supporting inhibitory regulation of fear expression [23].

Compared to estradiol, evidence for progesterone effects on extinction are less consistent. Although the aforementioned meta-analysis did not find an association between endogenous progesterone and extinction outcomes, women with lower progesterone levels showed significantly higher return of fear than those with higher progesterone levels, as supported by two studies [24, 25]. However, of these two studies, only one considered menstrual cycle phase [25], investigating the early follicular and mid-luteal phase, limiting insight into phase-specific effects.

Failing to account for menstrual cycle phase is an important limitation as effects of ovarian hormones on affective and cognitive processes may vary substantially between and within hormone-sensitive individuals, as proposed by the Dimensional Affective Sensitivity to Hormones across the Menstrual Cycle (DASH-MC) framework [28]. Specifically, although not all individuals experience such cycle-phase-related changes, a subset of hormone-sensitive individuals may show heightened affective reactivity to different hormonal changes across the menstrual cycle, which may explain heterogeneity between and within studies. Three dimensions have been proposed, with individuals showing (1) luteal-onset negative affect (e.g., irritability), which is most likely driven by sensitivity to estradiol and progesterone surges; (2) perimenstrual-onset negative affect (e.g., depressed mood), most likely triggered by sensitivity to low or falling estradiol; and/or (3) periovulatory-onset positive affect (e.g., risky reward seeking behavior), most likely driven by sensitivity to estradiol surges.

Compared to naturally cycling individuals, individuals using hormonal contraceptives (HCs), which suppress endogenous estradiol and progesterone synthesis, have demonstrated impaired extinction recall [29–32]. This effect has been observed across different HC formulations, including three studies providing evidence for users of combined monophasic oral contraceptives [29, 31, 32] and one study providing evidence for a mixed sample using different types of HCs (e.g., oral contraceptives, vaginal rings, intrauterine devices (IUDs), and arm implants) [30]. However, these studies did not specifically assess menstrual cycle phase among naturally cycling women, limiting interpretations about cycle phase as well as cycle-phase-specific comparisons with individuals using HCs.

Given that endogenous estradiol and progesterone fluctuate across the menstrual cycle, it has been proposed that the timing of exposure-based interventions may significantly impact treatment outcomes [20]. Rodent studies demonstrated that extinction learning may be enhanced during menstrual cycle phases marked by elevated endogenous estradiol and progesterone levels [33]. However, no study has yet investigated the effect of standardized, biologically informed menstrual cycle phases on exposure therapy outcomes. Recent work has emphasized the role of the menstrual cycle and ovarian hormones in the development, maintenance, and treatment of mental health conditions, highlighting important possibilities for personalized psychotherapy approaches [34]. This emerging perspective underscores the relevance of considering menstrual cycle related hormonal context in psychotherapeutic research.

The present study, therefore, aimed to investigate the effects of menstrual cycle phase, HCs, and endogenous ovarian hormones on the day of exposure therapy on treatment outcomes in women with spider phobia. Participants with symptoms of spider phobia were recruited to undergo a single session of exposure therapy. Women underwent exposure therapy either during the early to mid-follicular phase, the mid-luteal phase, or as users of HCs. The following hypotheses were tested: (a) women in the mid-luteal phase on the day of exposure-based cognitive behavioral therapy (CBT) would show a greater reduction in behavioral avoidance and phobic fear compared to those in the early to mid-follicular phase and those using HC; and (b) the higher the endogenous estradiol concentrations on the day of exposure-based CBT, the greater the reduction in behavioral avoidance and phobic fear. Exploratory analyses further examined whether endogenous progesterone, the progesterone-to-estradiol ratio, testosterone, and positive affect on the day of exposure therapy were associated with reductions in behavioral avoidance and phobic fear. The study was preregistered on the Open Science Framework (OSF; osf.io/tfp5n).

## Methods and Materials

This study was conducted as part of a larger research project which investigated the effects of endogenous hormones on exposure therapy success of patients with spider phobia. As compensation, each participant received vouchers for restaurants with a total value of 80 CHF.

### Sample

The study sample consisted of individuals who had registered for exposure therapy to overcome their fear of spiders at the Outpatient Clinic for CBT and Behavioral Medicine of the University of Zurich. Participants were contacted by study investigator 1 to be given information about both the group therapy and the study. Interested participants completed a telephone screening interview to assess eligibility based on the inclusion and exclusion criteria.

The inclusion criteria for the larger study encompassed women and men aged 18–65 years, with good German language skills, a body mass index between 18 and 30, and symptoms of arachnophobia, as assessed using a structured clinical interview. In addition, women had to have a regular menstrual cycle (on average between 21 and 37 days with 2–3 days of variability), use HCs (not restricted to a specific type, given previous findings [30]), or be postmenopausal as assessed by the Stages of Reproductive Aging Workshop (STRAW) criteria [35].

Exclusion criteria included current pregnancy, breastfeeding, or comorbid physical illnesses affecting hormone balance (e.g., polycystic ovary syndrome). A further exclusion criterion was perimenopausal stage, based on self-reported menstrual bleeding patterns as defined by the STRAW criteria [35]. Specifically, participants were excluded if they reported no natural bleeding within the past 12 months, changes in cycle length of 7 days or more, or temporary cessation of menstruation for 2 months or longer compared with earlier regular cycles. Perimenopausal status was exclusionary given the increased variability in ovarian hormone fluctuations and irregularity in menstrual cycle length, which preclude reliable determination of menstrual cycle phases [36]. Further exclusion criteria were lifetime comorbid psychotic disorders, bipolar disorders, current depressive episodes/disorders, substance abuse (last 2 years), drug dependence, eating disorders (last 5 years), use of psychoactive substances (last 2 months), and current use of hormones or medications that affect the endocrine system (except HCs). Participants who were currently undergoing psychotherapeutic or psychiatric treatment or who planned to undergo such treatment during the 3-month study phase or to take medication affecting the endocrine system were excluded. Participants who had registered for the present exposure therapy for a second time were also excluded.

The participant flowchart is displayed in Figure 1. Of the N = 55 enrolled participants, 10 were not included in analyses for various reasons: two participants could not be contacted for the posttreatment and follow-up and were, therefore, considered dropouts, three were men, two reported postmenopausal status, two were in the periovulatory phase on the day of exposure therapy (not enough participants to conduct meaningful group-level analyses of periovulatory effects), and one had insufficient information to determine menstrual cycle phase during exposure therapy (i.e., no positive ovulation test and cycle longer than 37 days). Although inclusion required clinically relevant spider phobia symptoms rather than a formal diagnosis, all but one participant met diagnostic criteria. This participant reported intense panic responses to spiders but no avoidance or daily impairment and was retained based on clinical judgment. The final sample, therefore, consisted of *n* = 45 participants. Of these, *n* = 15 used HCs and *n* = 30 were naturally cycling. Among naturally cycling women, *n* = 15 were in the early to mid-follicular phase, and *n* = 15 were in the mid-luteal phase on the day of exposure therapy. Regarding HC use, 40% reported taking combined monophasic oral contraceptive pills (duration range 1–10 years) with varying types and doses of synthetic estrogen and progestin, 46.6% reported an IUD (duration range 1–3 years) containing the progestin levonorgestrel (LNG), 6.7% reported a vaginal ring containing both a synthetic estrogen and a progestin, and 6.7% reported a subdermal implant containing progestin only (duration range 1–8 years).

**Fig. 1.**
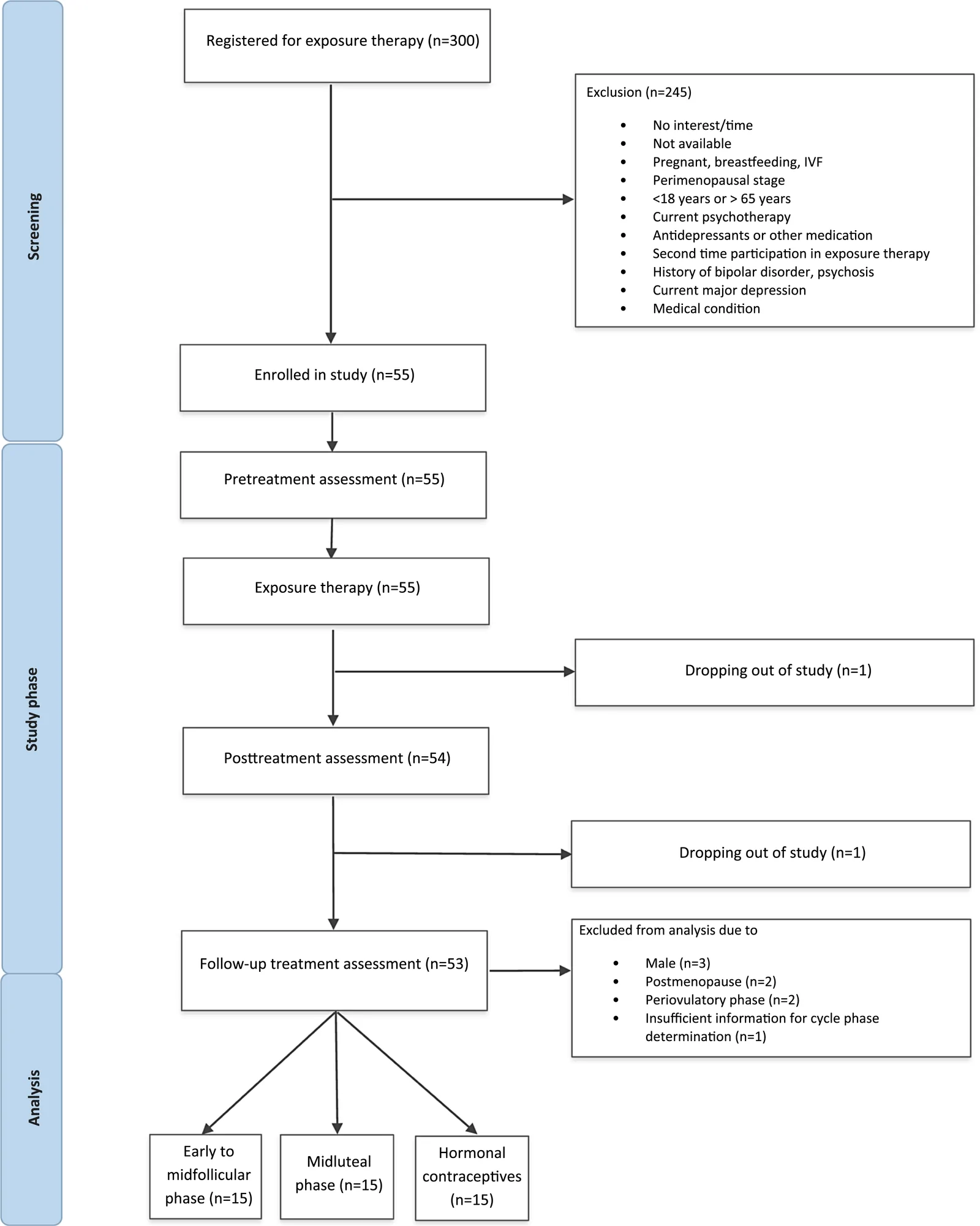
Flowchart.

### Procedure

The study design and procedure are illustrated in Figure 2. Participation after screening included four appointments: an initial pretreatment assessment, exposure therapy session, posttreatment assessment, and follow-up assessment. The pretreatment assessment took place during the week before the exposure therapy. After written informed consent, sociodemographic and lifestyle information, and medical history were obtained by forms, spider phobia was then assessed with the Structured Clinical Interview for the Diagnostic and Statistical Manual of Mental Disorders IV (SCID-IV) [37]. Further, the Fear of Spiders Questionnaire (FSQ) and the Spider Phobia Questionnaire (SPQ) were administered, and the Behavioral Avoidance Test (BAT) was conducted (see below). Participants were then provided a set of questionnaires assessing control variables to complete at home.

**Fig. 2.**
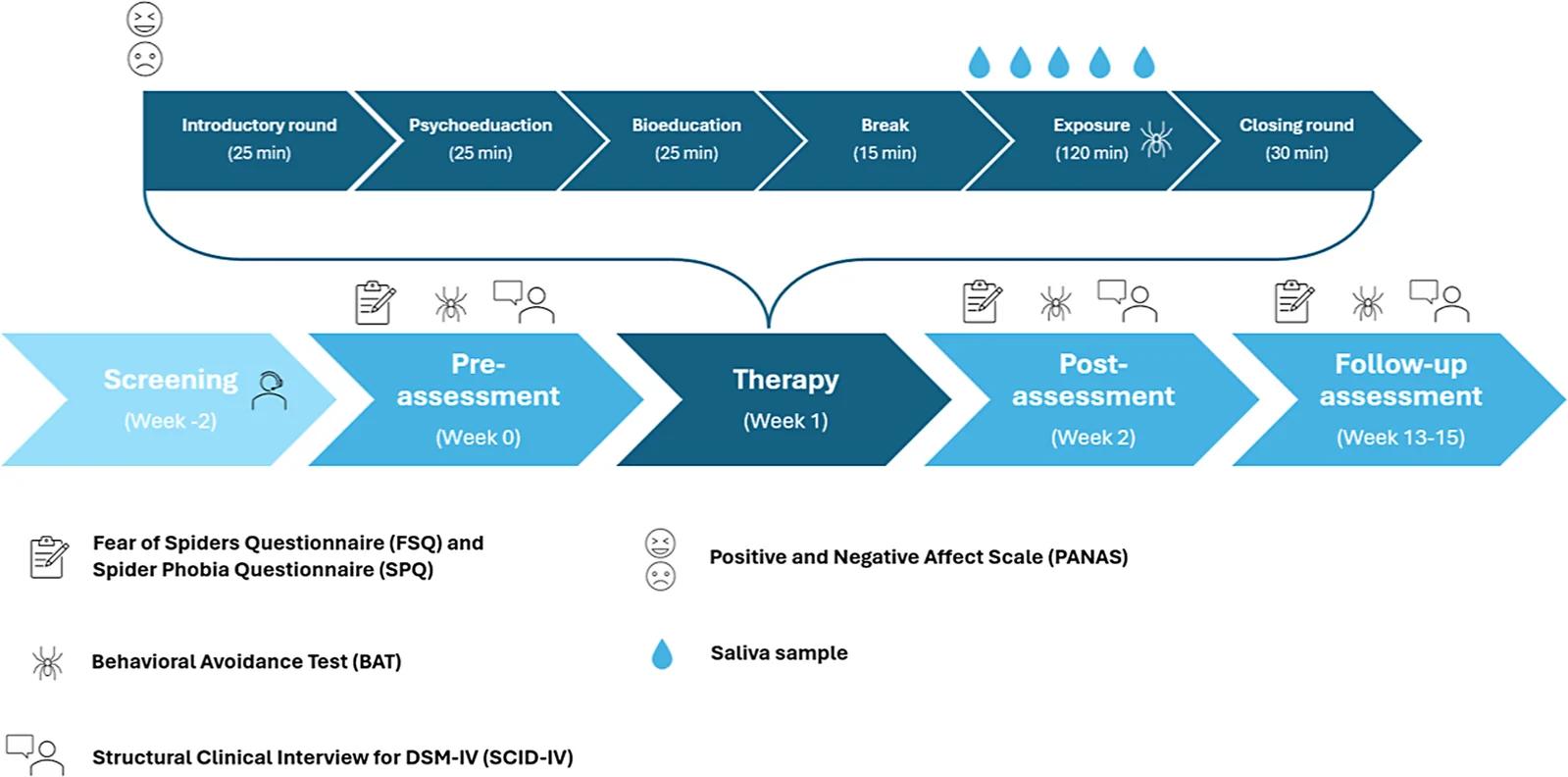
Study design. The study procedure included screening, pretreatment assessment, exposure therapy, 1-week posttreatment assessment, and 3-month follow-up. At pretreatment, posttreatment, and follow-up assessments, phobic fear of spiders was measured using the Fear of Spiders Questionnaire (FSQ) and the Spider Phobia Questionnaire (SPQ). Avoidance behavior toward a spider was assessed using a behavioral approach test. Moreover, a structured clinical interview was conducted to assess spider phobia diagnosis (Structural Clinical Interview for DSM-IV). Exposure therapy consisted of a single 4-h group-based CBT session, beginning with an introductory round, followed by psychoeducation, bioeducation, individual exposure to spiders, and ending with a closing round.

Exposure therapy started either at 08.00 a.m. or 05.00 p.m. Upon arrival, study investigator 3 received the questionnaire set from the participants and asked them to fill in the Positive and Negative Affect Schedule (PANAS) [38]. The therapy consisted of a single 4 h session of an exposure-based CBT program, beginning with group therapy and followed by individual exposures. Between May 2021 and February 2025, a total of 30 sessions were conducted, each including ten participants, of whom one to four were enrolled in the study. Each group was heterogeneous with respect to gender and hormonal and menstrual cycle phase status. Group therapy started with a 25 min introductory round, in which individual experiences with spiders, triggers, and traumatic experiences were exchanged, and individual treatment goals were formulated. This was followed by a 25 min psychoeducation about fear and anxiety disorders, and information about the mechanisms behind exposure to phobic stimuli. These elements were guided by two clinical psychologists (both women), who were trained in exposure-based group CBT by UG and GP. A biologist/curator of a local zoo then delivered a 25 min bio-education about spiders as well as popular myths about spiders. After a break of 15 min, participants were individually exposed to spiders of different sizes for 120 min, which they approached while being supervised by the two therapists. During this time, exposure steps were rated by study investigator 3. The patient moved to the next step when their distress levels reduced by at least 50%. Only spiders indigenous to Switzerland were used (size range: 1–5cm). Moreover, during the exposition, five saliva samples were collected (at the beginning, +30 min, +60 min, +90 min, +120 min). The therapy session ended with a 30 min closing round. All biological samples were immediately stored at −20°C until further biochemical analysis. The posttreatment assessment took place 1 week after the therapy session and involved participants completing the SCID, FSQ and SPQ questionnaires, and the BAT again. During the follow-up assessment, around 12–14 weeks after the therapy session, the same posttreatment assessment procedures were repeated. At the end of this appointment, all participants were fully debriefed about all study aims. The participants, clinical psychologists, study investigators 2 and 3, and biologist conducting the biochemical analysis were blind to the study hypotheses.

### Measures

*Affect* was assessed immediately before the therapy session via the German version of the Positive and Negative Affect Schedule (PANAS) [38, 39]. This 20-item scale measures two dimensions of affect: positive and negative affect. The instructions are as follows: “Indicate to what extent you feel this way right now, that is, at the present moment,” followed by ten adjectives for each subscale (e.g., “distressed”). Each item is rated on a 5-point Likert scale ranging from 1 (“not at all”) to 5 (“extremely”).

*Behavioral avoidance* was assessed using a behavioral approach test (BAT). In brief, the BAT used a live 5 cm house spider and tested participants’ ability to approach it. Participant behavior is rated on a 10-point scale: 0 = refuses to enter the test room, 1 = stops 3 m from the container, 2 = stops 2 m from the container, 3 = stops 1 m from the container, 4 = stops close to the windowsill with the container, 5 = touches the container, 6 = removes the lid, 7 = puts a hand in the container, 8 = touches the inside of the container, 9 = touches the spider with one finger.

*Phobic fear* was assessed via the German versions of the Fear of Spiders Questionnaire (FSQ) [40, 41] and the Spider Phobia Questionnaire (SPQ) [41, 42]. The FSQ measures the severity of phobic fear using 18 items rated on a 7-point Likert scale ranging from 0 (“does not apply to me at all”) to 6 (“fully applies to me”). The SPQ measures phobic fear via 43 items that are answered by checking either “yes” or “no.” The total score of the SPQ reaches up to 33 points, with higher scores indicating greater fear of spiders [41]. Both questionnaires were administered at three time points: pretreatment, posttreatment, and follow-up assessment.

### Hormonal Assessment

Saliva samples were collected using SaliCaps (IBL, Hamburg, Germany). Estradiol, progesterone, testosterone, and cortisol were analyzed from the first saliva sample collected during the exposure therapy, consistent with prior evidence linking hormone levels during exposure-based learning to subsequent treatment outcomes [30, 43, 44]. All biochemical analyses were conducted at the biochemical laboratory of the Institute of Psychology at the University of Zurich using commercially available luminescence immunoassays (IBL, Hamburg, Germany). The intra- and inter-assay variance of these kits was < 10%.

### Menstrual Cycle Assessment

Menstrual cycle phase was assessed based on best practice recommendations by Schmalenberger et al. [45]. Participants reported their average cycle length and typical variability between cycles. Menstrual cycle phase on the day of exposure therapy was determined based on the reported onset of the last menses prior to therapy and the onset of the subsequent menses. Participants were separated into two different groups based on the phase of their menstrual cycle on the day of therapy. The first group represented women in the early to mid-follicular phase, determined using the forward-counting method, defined as days +1 to +7 from the onset of menstrual bleeding, with the first day of bleeding designated as day +1. The second group represented women in the mid-luteal phase, determined using a combination of the forward-counting method and a luteinizing hormone (LH) ovulation test (Inopharm, Bern, Switzerland, threshold 20 mIU/mL), defined as days +5 to +11 following a positive LH test. The early to mid-follicular phase was selected because it is characterized by relatively low and stable endogenous estradiol and progesterone levels, whereas the mid-luteal phase was chosen to represent a hormonal milieu with comparatively higher levels of both hormones [45]. Of the 15 participants in the mid-luteal phase, ten obtained a positive LH test, whereas five did not. For participants without a positive LH test, ovulation was estimated retrospectively using the backward-counting method based on the reported onset of the subsequent menses, assuming a cycle length of 21–37 days. Participants using HCs formed a separate group. For users of cyclically administered HCs (combined oral pills or vaginal ring), exposure therapy was scheduled independent of intake phase (active vs. inactive), which was not assessed.

### Statistical Analysis

One-way analysis of variance (ANOVA) and Fisher’s exact tests were used to analyze group differences in baseline sample characteristics. For the first hypothesis, linear mixed-effects models with the within-subjects factor of assessment time (T1: pretreatment assessment, T2: 1-week posttreatment assessment, T3: 12–14 weeks follow-up assessment), the between-subjects factor of menstrual cycle group (early to mid-follicular phase, mid-luteal phase, and HC), and random intercepts for participants were used to assess changes in behavioral avoidance and phobic fear according to group status. Fixed effects were evaluated using Type III ANOVA-based *F* tests with Satterthwaite-approximated degrees of freedom. Significant main and interaction effects, as well as those reaching trend-level significance (*p* < 0.1), were followed up using planned contrasts based on estimated marginal means. For significant main effects, pairwise comparisons examined differences between factor levels. For significant interaction effects, planned within-group contrasts tested changes from pretreatment to posttreatment assessment (pre-post) and from pretreatment to follow-up (pre-FU) assessment, and between-group contrasts compared these change scores across groups.

For the second hypothesis, Spearman rank correlations were conducted to investigate associations between estradiol levels and pre-post and pre-FU changes in behavioral avoidance and phobic fear. As an exploratory extension and for comparability with prior work [30, 46], additional linear mixed-effects models were conducted using median-split hormone groups (i.e., estradiol, progesterone, the progesterone-to-estradiol ratio, and testosterone) on the day of exposure therapy as between-subjects factors. Exploratory Spearman rank correlations were also conducted for progesterone, progesterone-to-estradiol ratio, and testosterone to investigate correlation with changes in behavioral avoidance and phobic fear. To account for potential confounding, baseline phobia severity and positive affect on the day of exposure were added as covariates in all models in a second exploratory step, given evidence that higher positive affect may facilitate exposure therapy outcomes [47]. Because positive affect may itself vary across the menstrual cycle [48] and may thus represent a potentially meaningful explanatory variable, we additionally conducted exploratory analyses treating positive affect on the day of exposure therapy as a predictor of treatment response.

Model assumptions were tested a priori. Although behavioral avoidance was measured on an ordinal scale, it was treated as a continuous variable in all analyses, consistent with previous studies [30, 32, 44]. This specification was further supported by the number of ordered categories, distribution of responses, and model residuals. Both menstrual cycle phase and hormone levels were analyzed in order to capture complementary aspects. While cycle phase is a convenient estimate of hormone levels, direct hormone measurements capture individual differences that phase categorizations may miss. Further, phase-based models allow examining differences across clinically meaningful hormonal contexts, while hormone-based analyses enable the examination of continuous hormone concentrations independent of phase classification. The Benjamini-Hochberg method was used to correct for multiple testing [49]. All analyses were conducted in R (Version 4.4.5) [50].

### Power Analysis

An a priori power analysis was conducted using G*Power [51] to determine the required sample size for the planned analyses. Based on a previous similar study [30] reporting a moderate effect size (e.g., *η*p^2^ = 0.10), the power analysis (α = 0.05, power = 80%) indicated that a total sample size of N = 60 participants would be required to detect the interaction between time and menstrual cycle phase groups in a mixed ANOVA. The preregistration specified a larger target sample size (N = 120) because the study was designed to test additional hypotheses and outcomes beyond those examined in the present manuscript.

## Results

### Sample Characteristics

Sample characteristics are displayed in Table 1. Only progesterone differed significantly between the menstrual cycle groups, with women in the mid-luteal phase showing higher endogenous levels than those in the early to mid-follicular phase (*M* difference = 35.4, 95% CI [4.6, 66.2], *p* = 0.021) and those using HCs (*M* difference = 29.7, 95% CI [0.99, 60.6], *p* = 0.059). Moreover, a trend emerged for progesterone-to-estradiol ratio group differences, with post hoc tests indicating a trend for women in the mid-luteal phase to show a higher ratio than those in the early to mid-follicular groups (*M* difference = 12.5, 95% CI [−0.63, 25.5], *p* = 0.065). Within the HC group, endogenous estradiol levels were comparable between participants using oral contraceptives (*M* = 3.1, *SD* = 3.2) and those using other HC types (*M* = 3.0, *SD* = 1.2). However, participants using oral contraceptives showed descriptively lower endogenous progesterone levels (*M* = 19.2, *SD* = 10.9) than those using other HC types (*M* = 44.4, *SD* = 42.9). Distribution of menstrual cycle groups to median split groups of hormones are illustrated in Figure 3. Menstrual cycle groups did not significantly differ in negative affect on the day of exposure therapy, nor in baseline phobic fear or behavioral avoidance as assessed 1 week prior to the exposure therapy. All but one participant fulfilled criteria for spider phobia at pretreatment assessment (*n* = 44). This number decreased to 22 at follow-up, corresponding to around half of the sample no longer meeting criteria for specific phobia in past month.

**Table 1.** Sample characteristics

Variable	Total sample (*n* = 45)	Early to mid-follicular phase (*n* = 15)	Mid-luteal phase (*n* = 15)	HCs (*n* = 15)	Statistics, *p* value
	M (SD) or N (%)	M (SD) or N (%)	M (SD) or N (%)	M (SD) or N (%)	
Age (years)	29.3 (7.4)	30.1 (7.8)	30.9 (6.8)	27.1 (7.7)	F(2,42) = 1.07, *p* = 0.349
Body mass index	22.6 (3.5)	21.6 (1.6)	22.9 (4.1)	23.6 (4.1)	F(2,42) = 1.29, *p* = 0.284
Education					Fisher’s test, *p* = 0.418
No school diploma	1 (2.2%)	–	1 (6.7%)	–	
Compulsory school	2 (4.4%)	–	–	2(13.3%)	
Vocational school	2 (4.4%)	–	1 (6.7%)	1 (6.7%)	
High school diploma	13 (28.8%)	5 (33.3%)	4 (26.7%)	4 (26.7%)	
Bachelor’s degree	11 (24.4%)	2 (13.3%)	5 (33.3%)	4 (26.7%)	
Master’s degree	12 (26.6%)	6 (40%)	4 (26.7%)	2 (13.3%)	
Doctorate	1 (2.2%)	1 (6.7%)	–	–	
Other	3 (6.6%)	1 (6.7%)	–	2 (13.3%)	
Specific phobia criteria^a^					Fisher’s test, *p* = 0.484
Mild	12 (26.6%)	5 (33.3%)	5 (33.3%)	2 (13.3%)	
Moderate	26 (57.7%)	8 (53.3%)	7 (46.7%)	11 (73.3%)	
Severe	6 (13.3%)	1 (6.6%)	3 (20%)	2 13.3%)	
Not fulfilled	1 (2.2%)	1 (6.6%)	–	–	
PANAS^b^					
Positive affect	39.3 (6.1)	40.5 (6.3)	38.3 (6.9)	39.0 (5.5)	F(2,32) = 0.36, *p* = 0.696
Negative affect	29.4 (3.2)	29.7 (2.2)	30.3 (3.9)	28.4 (3.5)	F(2,32) = 0.99, *p* = 0.380
Baseline behavioral avoidance (BAT)	3.4 (2.2)	4.1 (2.4)	3.5 (2.3)	2.5 (1.8)	F(2,42) = 2.01, *p* = 0.150
Baseline phobic fear					
FSQ^c^	73.3 (17.4)	67.1 (19.5)	72.5 (18.9)	80.3 (10.8)	F(2,42) = 2.29, *p* = 0.113
SPQ^d^	23.0 (4.3)	21.1 (5.5)	24.0 (3.5)	23.9 (3.1)	F(2,42) = 2.38, *p* = 0.100
Daytime of exposure therapy					Fisher’s test, *p* = 0.648
Morning (8 AM)	19 (42.2%)	5 (33.3%)	6 (40.0%)	8 (53.3%)	
Evening (5 PM)	26 (57.8%)	10 (66.7%)	9 (60.0%	7 (46.7%)	
Estradiol, pg/mL	2.8 (1.7)	2.7 (2.2)	2.8 (1.0)	3.0 (1.9)	F(2,36) = 0.09, *p* = 0.907
Progesterone, pg/mL	40.8 (35.2)	28.7 (21.6)	64.1 (39.8)	34.3 (35.4)	F(2,39) = 4.39, *p* = 0.019
Testosterone, pg/mL	9.1 (7.8)	11.2 (10.6)	7.4 (3.9)	8.5 (7.3)	F(2,28) = 0.64, *p* = 0.534
Progesterone to estradiol ratio	17.8 (14.6)	13.8 (9.8)	26.2 (18.4)	14.6 (12.8)	F(2,42) = 1.07, *p* = 0.053
Cortisol	2.0 (3.3)	2.6 (4.8)	1.24 (1.2)	2.03 (2.5)	F(2,39) = 0.56, *p* = 0.575

BAT, behavioral avoidance test.

^a^ Assessed with Structured Clinical Interview for the Diagnostic and Statistical Manual of Mental Disorders (SCID) during the pretreatment assessment.

^b^ Positive and Negative Affect Scale (range: 10–50).

^c^ Fear of Spiders Questionnaire (range: 0–108).

^d^ Spider Phobia Questionnaire (range: 0–33).

**Fig. 3.**
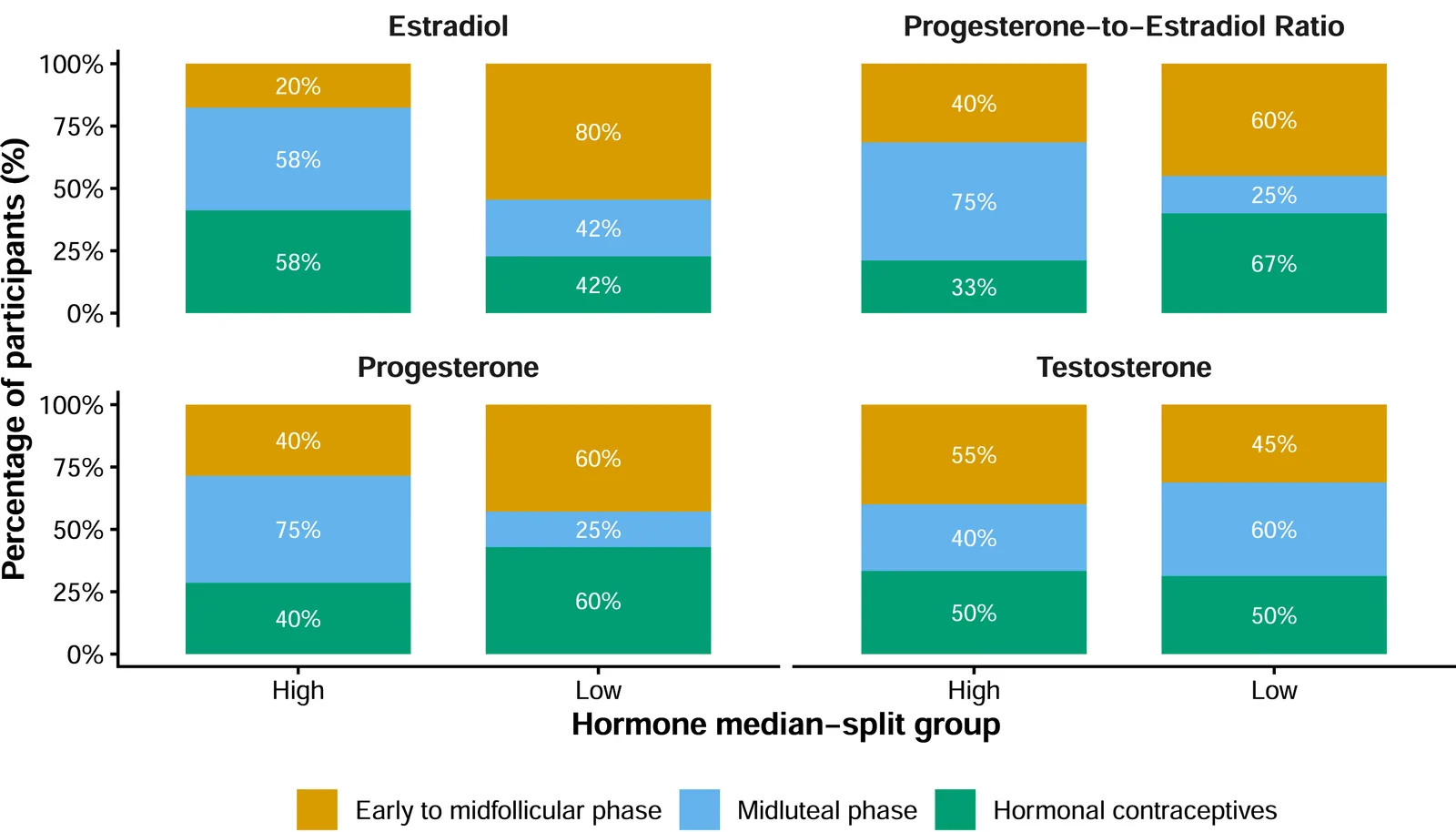
Distribution of menstrual cycle groups across hormone median split categories. Bars represent the percentage of participants within each hormone median-split category (low vs. high). Median split groups were calculated across the full sample based hormone median cut-offs of 3.0 pg/mL for estradiol, 30.1 pg/mL for progesterone, 12.1 for the progesterone-to-estradiol ratio, and 7.7 pg/mL for testosterone.

### Behavioral Avoidance

Mixed model ANOVA results for behavioral avoidance according to menstrual cycle group and median split groups of hormones are displayed in Table 2.

**Table 2.** Mixed ANOVA results for behavioral avoidance and phobic fear according to menstrual cycle group and median split groups of hormones

Variable	Time effect				Group effect				Interaction effect (time × group)			
	*F*	*df*	*p* value	*ηp* ^*2*^	*F*	*df*	*p* value	*ηp* ^*2*^	*F*	*df*	*p* value	*ηp* ^*2*^
Behavioral Avoidance Test (BAT)												
Menstrual cycle group	127.99	2.89	<0.001*	0.74	2.79	2.45	0.071	0.11	1.27	4.89	0.284	0.05
Estradiol group	97.11	2.77	<0.001*	0.72	5.75	1.39	0.021*	0.13	1.62	2.77	0.202	0.04
Progesterone group	114.61	2.83	<0.001*	0.73	0.13	1.42	0.711	0.003	3.02	2.83	0.054	0.07
Progesterone-to-estradiol ratio group	106.70	2.77	<0.001*	0.73	0.45	1.39	0.504	0.01	3.54	2.77	0.033	0.08
Testosterone group	78.97	2.61	<0.001*	0.72	0.0001	1.31	0.991	<0.001	0.78	2.61	0.459	0.03
Positive affect group	87.84	2.69	<0.001*	0.72	1.30	1.35	0.260	0.04	0.19	2.69	0.826	0.005
Fear of Spiders Questionnaire (FSQ)												
Menstrual cycle group	102.86	2.90	<0.001*	0.70	1.06	2.45	0.352	0.05	0.37	4.90	0.825	0.02
Estradiol group	90.54	2.78	<0.001*	0.70	4.97	1.39	0.031*	0.11	0.20	2.78	0.814	0.005
Progesterone group	94.21	2.84	<0.001*	0.69	0.45	1.42	0.504	0.01	0.85	2.84	0.429	0.02
Progesterone-to-estradiol ratio group	92.66	2.78	<0.001*	0.70	0.02	1.39	0.872	0.0006	0.11	2.78	0.892	0.002
Testosterone group	82.85	2.62	<0.001*	0.73	0.06	1.31	0.799	0.003	0.38	2.62	0.681	0.01
Positive affect group	78.88	2.70	<0.001*	0.69	6.46	1.35	0.015*	0.16	0.84	2.70	0.432	0.02
Spider Phobia Questionnaire (SPQ)												
Menstrual cycle group	64.56	2.90	<0.001*	0.59	0.92	2.45	0.403	0.04	0.91	4.90	0.461	0.04
Estradiol group	65.22	2.78	<0.001*	0.63	5.09	1.39	0.029*	0.12	1.64	2.78	0.199	0.04
Progesterone group	66.71	2.84	<0.001*	0.61	1.03	1.42	0.315	0.02	1.81	2.84	0.169	0.04
Progesterone-to-estradiol ratio group	65.22	2.78	<0.001*	0.63	0.06	1.39	0.796	<0.001	0.53	2.78	0.589	0.01
Testosterone group	60.73	2.62	<0.001*	0.66	0.26	1.31	0.610	0.008	2.75	2.62	0.071	0.08
Positive affect group	49.27	2.70	<0.001*	0.58	9.20	1.35	0.004*	0.21	1.02	2.70	0.364	0.03

Hormone median split groups were calculated across the full sample with hormone median cut-offs of 3.0 pg/mL for estradiol, 30.1 pg/mL for progesterone, 12.1 for the progesterone-to-estradiol ratio, and 7.7 pg/mL for testosterone and positive affect median split group with cut-off of 41. *Significant after correction for multiple testing.

#### Menstrual Cycle

Changes in behavioral avoidance according to menstrual cycle group are displayed in Figure 4a. After correction for multiple testing, behavioral avoidance significantly improved over time, with planned post hoc contrasts revealing a significant decrease in all groups (pre-post: *b* = −2.76, *SE* = 0.21, 95% CI [−3.19, −2.32], *t* = −12.63, *p* < 0.001; pre-FU: *b* = −3.08, *SE* = 0.22, 95% CI [−3.51, −2.64], *t* = −13.98, *p* < 0.001; post-FU: *b* = −0.32, *SE* = 0.22, 95% CI [−0.75, 0.11], *t* = −1.45, *p* = 0.148). Moreover, a trend for a main effect of menstrual cycle group was observed, suggesting overall behavioral avoidance differences based on cycle phase across assessment time points. Exploratory post hoc contrasts revealed a trend toward lower behavioral avoidance in the early to mid-follicular group compared to the HC group (*b* = 1.62, *SE* = 0.73, 95% CI [−0.18, 3.43], *t* = 2.22, *p* = 0.077). However, no significant interaction of time and menstrual cycle group emerged, indicating an absence of group differences in changes in behavioral avoidance over time. Results did not substantially change after controlling for baseline phobia severity and positive affect on the day of exposure therapy.

**Fig. 4.**
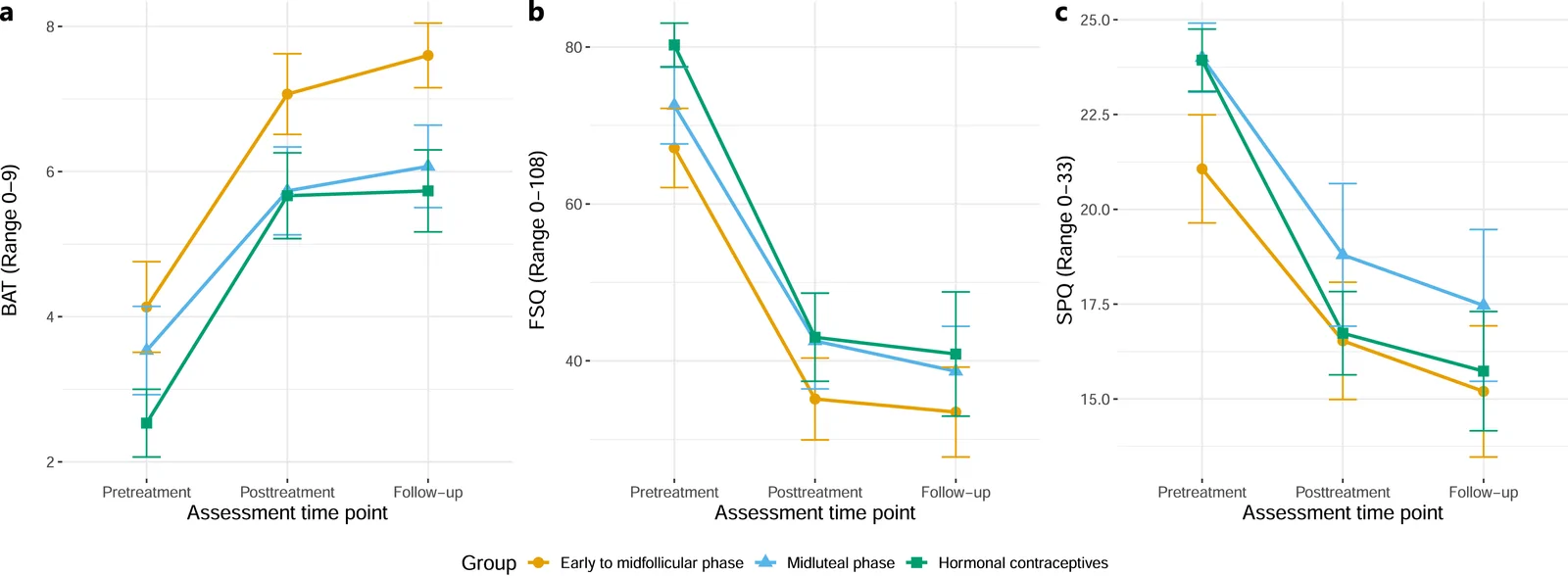
Behavioral avoidance and self-reported phobic fear across pretreatment, posttreatment, and follow-up assessment by menstrual cycle group status. **a** Behavioral avoidance, with higher values indicating lower avoidance. **b** Self-reported phobic fear assessed with the Fear of Spiders Questionnaire (FSQ), with lower scores indicating lower fear. **c** Self-reported phobic fear assessed with the Spider Fear Questionnaire (SPQ), with lower scores indicating lower fear. Data are shown for participants in the early to mid-follicular phase, mid-luteal phase, and hormonal contraception (HC) groups at baseline, 1-week posttreatment, and 12–14 weeks follow-up.

#### Estradiol

Contrary to expectation, correlation analyses indicated that estradiol was not associated with pre-post or pre-FU behavioral avoidance changes (see Table 3), which remained unchanged in linear regression analyses accounting for phobia severity and positive affect.

**Table 3.** Spearman correlation results for behavioral avoidance and phobic fear

Variable	Pre- to posttreatment		Pretreatment to follow-up	
	*ρ*[95% CI]	*p* value	*ρ*[95% CI]	*p* value
Behavioral Avoidance Test (BAT)				
Estradiol	−0.26[−0.53.0.05]	0.098	−0.21 [−0.50.0.11]	0.189
Progesterone	−0.49 [−0.69.0.22]	0.001*	−0.33 [−0.57,−0.02]	0.035
Progesterone-to-estradiol ratio	−0.38 [−0.61,−0.07]	0.018*	−0.20 [−0.49.0.12]	0.218
Testosterone	−0.006 [−0.35.0.34]	0.975	0.24 [−0.12.0.55]	0.191
Positive affect	−0.16 [−0.34.0.31]	0.927	−0.15 [−0.47.0.18]	0.369
Fear of Spiders Questionnaire (FSQ)				
Estradiol	0.004 [−0.31.0.31]	0.980	−0.03 [−0.34.0.28]	0.840
Progesterone	<0.001 [−0.30.0.30]	0.998	−0.15 [−0.44.0.15]	0.318
Progesterone-to-estradiol ratio	0.09 [−0.22.0.40]	0.547	0.05 [−0.26.0.36]	0.723
Testosterone	0.009 [−0.34.0.36]	0.960	−0.12 [−0.45.0.24]	0.501
Positive affect	−0.27 [−0.55.0.06]	0.112	0.03 [−0.30.0.36]	0.841
Spider Phobia Questionnaire (SPQ)				
Estradiol	−0.08 [−0.39.0.23]	0.598	0.18 [−0.14.0.47]	0.263
Progesterone	−0.12 [−0.40.0.19]	0.463	−0.20 [−0.48.0.10]	0.192
Progesterone-to-estradiol ratio	0.04 [−0.27.0.35]	0.806	−0.12 [−0.42.0.20]	0.463
Testosterone	0.08 [−0.27.0.42]	0.637	0.26 [−0.56.0.09]	0.150
Positive affect	−0.33 [−0.59.0.002]	0.052	−0.09 [−0.41.0.24]	0.575

For behavioral avoidance, a positive correlation score indicates that higher predictor values are associated with greater reductions in avoidance over time (improvement), whereas a negative correlation worsening. For phobic fear (both FSQ and SPQ), a positive correlation indicates that higher predictor values are associated with greater fear over time (worsening), whereas a negative correlation indicates improvement. Positive affect was assessed with the positive affect subscale of the Positive and Negative Affect Scale (PANAS). 95% confidence intervals were estimated using Fisher’s z-transformation.

*Significant after correction for multiple testing.

Exploratory follow-up analyses using median split groups of estradiol revealed a significant main effect of group, indicating overall behavioral avoidance differences between estradiol groups. Post hoc comparisons demonstrated that the high estradiol group showed greater behavioral avoidance than the low estradiol group (difference = −1.46, *SE* = 0.62, 95% CI [−2.72, −0.19], *t* = −2.33, *p* = 0.024). However, no interaction effect between time and estradiol group was observed. These results remained significant after correction for multiple testing and did not substantially change after controlling for phobia severity and positive affect.

#### Progesterone

Exploratory correlation analyses revealed that lower progesterone levels were associated with a greater improvement in pre-post and pre-FU behavioral avoidance (see Table 3). However, only the pre-post association remained significant after correction for multiple testing. This pattern remained unchanged in linear regression analyses additionally adjusting for phobia severity and positive affect.

Further exploratory analyses using median split groups of progesterone indicated no main effect of progesterone group on behavioral avoidance. However, a trend for an interaction between progesterone group and time emerged (see online suppl. Fig. S1; for all online suppl. material, see https://doi.org/10.1159/pps/aemag012), which turned significant after controlling for phobia severity and positive affect (*F*(2,65) = 5.10, *p* = 0.008, *η*p^2^ = 0.14). Post hoc between-group contrasts indicated that both groups showed pre-post and pre-FU improvement; however, the magnitude of improvement differed between groups: women with high progesterone levels showed a reduced improvement compared to women with low progesterone levels from pretreatment to posttreatment assessment (*b* = 1.05, *SE* = 0.44, 95% CI [0.2, 1.9], *t* = 2.37, *p* = 0.019), but not from pretreatment to follow-up assessment (*b* = 0.64, *SE* = 0.44, 95% CI [−0.2, 1.5], *t* = 1.44, *p* = 0.153).

#### Progesterone-To-Estradiol Ratio

Exploratory correlation analyses indicated that a lower progesterone-to-estradiol ratio was associated with a greater improvement in behavioral avoidance from pretreatment to posttreatment assessment but not from pretreatment to follow-up assessment (Table 3). Although this finding withstood correction for multiple testing, it was not observed in linear regression analyses adjusted for phobia severity and positive affect.

Exploratory analyses using median split groups of the ratio as the between-subject factor revealed a significant interaction between the progesterone-to-estradiol ratio group and time. Post hoc comparisons revealed that women with a high progesterone-to-estradiol ratio showed poorer improvement than those with a low ratio from pretreatment to posttreatment assessment (*b* = 1.15, *SE* = 0.44, 95% CI [0.25, 2.04], *t* = 2.56, *p* = 0.012), but not from pretreatment to follow-up assessment (*b* = 0.42, *SE* = 0.45, 95% CI [−0.47, 1.33], *t* = 0.94, *p* = 0.346). Although this interaction effect remained unchanged after controlling for positive affect and phobia severity, it did not withstand correction for multiple testing.

#### Testosterone

Exploratory analyses revealed no significant associations between testosterone and behavioral avoidance (Table 3). This pattern remained unchanged after accounting for phobia severity and positive affect.

#### Positive Affect

Exploratory analyses revealed no significant associations between positive affect and behavioral avoidance, across both correlation analyses (Table 3) and median split group comparisons, and results were unchanged after adjusting for phobia severity.

### Self-Reported Phobic Fear

Mixed model ANOVA results for phobic fear assessed with the FSQ and SPQ according to menstrual cycle group and median split groups of hormones are displayed in Table 2.

#### Menstrual Cycle

Changes in self-reported phobic fear assessed with the FSQ and SPQ according to menstrual cycle group status are displayed in Figure 4b-c. After correction for multiple testing, phobic fear assessed with the FSQ significantly improved over time. Planned post hoc contrasts revealed a significant decrease in phobic fear among all groups (pre-post: *b* = 33.09, *SE* = 2.87, 95% CI [27.4, 38.8], *t* = 11.53, *p* < 0.001; pre-FU: *b* = 35.64, *SE* = 2.87, 95% CI [29.9, 41.3], *t* = 12.42, *p* < 0.001; post-FU: *b* = 2.56, *SE* = 2.87, 95% CI [−3.14, 8.25], *t* = 0.89, *p* = 0.375). However, no main or interaction effect of menstrual cycle group was found. Similarly, phobic fear assessed with the SPQ decreased significantly over time, whereas no main or interaction effect of menstrual cycle group status was found. Planned post hoc contrasts revealed an improvement in phobic fear among all groups (pre-post: *b* = 5.64, *SE* = 0.66, 95% CI [4.3, 6.9], *t* = 8.45, *p* < 0.001; pre-FU: *b* = 6.87, *SE* = 0.66, 95% CI [5.5, 8.1], *t* = 10.29, *p* < 0.001; post-FU: *b* = 1.22, *SE* = 0.66, 95% CI [−0.1, 2.5], *t* = 1.83, *p* = 0.070). These results did remained unchanged after accounting for phobia severity and positive affect.

#### Hormones

Contrary to expectation, estradiol on the day of exposure therapy was not associated with pre-post or pre-FU changes in phobic fear (both FSQ and SPQ), and the same was true for progesterone, the progesterone-to-estradiol ratio, and testosterone (Table 3). The results remained unchanged after adjusting for phobia severity and positive affect.

Exploratory follow-up analyses using median split of estradiol revealed a main effect of estradiol on FSQ and SPQ, but no interaction effect. Post hoc comparisons indicated that participants in the high estradiol group showed overall greater phobic fear than those in the low estradiol group (FSQ: difference = 12.5, *SE* = 5.74, 95% CI [0.87, 24.1], *t* = 2.17, *p* = 0.035; SPQ: difference = 3.45, *SE* = 51.57, 95% CI [0.2, 6.6], *t* = 2.19, *p* = 0.033). Moreover, a trend emerged for an interaction between testosterone group and time for phobic fear assessed with the SPQ, with post hoc comparisons indicating that women with low testosterone showed poorer improvement than those with high testosterone from pretreatment to follow-up treatment (*b* = 2.82, *SE* = 1.49, 95% CI [−0.15, 5.79], *t* = 1.89, *p* = 0.063), but not from pretreatment to posttreatment (*b* = −0.21, *SE* = 1.49, 95% CI [-3.19, 2.76], *t* = −0.14, *p* = 0.884). No further main or interaction effects were found for any of the other hormonal median split groups and the results remained comparable after adjusting for covariates.

#### Positive Affect

Exploratory analyses showed no significant associations between positive affect and phobic fear assessed with the SPQ; however, a trend emerged for higher positive affect scores on the day of the exposure therapy to be associated with greater reductions in phobic fear assessed with the FSQ (Table 3). Although no interaction effect was observed, a main effect for the positive affect median split group emerged, which remained consistent after correction for multiple testing. Post hoc comparisons indicating overall higher phobic fear in the low positive affect group compared to the high positive affect group (FSQ: *b* = −14.9, *SE* = 6.05, 95% CI [-27.2, −2.68], *t* = −2.46, *p* = 0.018; SPQ: *b* = −4.6, *SE* = 1.5, 95% CI [-7.76, −1.44], *t* = −2.94, *p =* 0.005). However, this group effect did not remain significant after accounting for phobic fear severity (FSQ: *F*(1,34) = 1.41, *p* = 0.243, *ηp*^*2*^*= 0.04;* SPQ: *F*(1,34) = 3.54, *p* = 0.068, *ηp*^*2*^ = 0.09).

### Post Hoc Sensitivity Analysis

As an exploratory post hoc sensitivity analysis, analyses were repeated without the participant who did not fulfill criteria for specific phobia. The results remained unchanged.

## Discussion

The present findings indicate that a single CBT exposure therapy session improved both behavioral avoidance and self-reported phobic fear in women with spider phobia, which may partially be modulated by endogenous ovarian hormone levels on the day of exposure therapy. Specifically, higher progesterone levels on the day of exposure therapy were associated with a reduced improvement in behavioral avoidance, but not self-reported phobic fear.

Menstrual cycle status on the day of exposure was not associated with behavioral avoidance or self-reported fear improvement, either from pretreatment to posttreatment assessment or from pretreatment to follow-up assessment. These findings are consistent with previous exploratory work indicating that self-reported cycle phase was not associated with exposure therapy improvement [30], although self-reported cycle phase is prone to misclassifications [52]. To our knowledge, this is the first study to examine standardized menstrual cycle group differences in clinical exposure therapy outcomes. By contrast, prior studies using standardized menstrual cycle phase classifications have largely been limited to experimental fear extinction paradigms. For instance, one such study among women with posttraumatic stress disorder (PTSD) has observed differences between menstrual cycle groups, with women in the mid-luteal phase showing impaired retention of extinction learning compared to those in the early follicular phase [25]. Although the present study did not indicate that menstrual cycle groups differed in the magnitude of symptom improvement, the associated effect sizes were small. Given the limited sample size and correspondingly limited statistical power, this null finding should be interpreted cautiously, and replication in larger samples is warranted. Despite differences in memory content and clinical presentation, individuals with PTSD and specific phobias relative to healthy controls show overlapping alterations within the neuronal fear-extinction circuitry, including the hippocampus, amygdala, and vmPFC [27, 43]. For instance, neuroimaging studies indicate similar neuronal activation patterns in PTSD and specific phobia when confronted with fearful cues or phobia-specific cues [43]. As ovarian hormones exert their effects through binding to receptors that are widely distributed across these neuronal regions, cycle-dependent ovarian hormone states during fear extinction may differently modulate molecular mechanisms in the neuronal fear-circuitry, including synaptic plasticity, that are critical for extinction learning, ultimately contributing to similar improvements after extinction learning in different clinical samples [23, 27]. However, the extent to which these neuronal alterations represent shared transdiagnostic mechanisms or disorder-specific neurobiological processes remains unclear, pointing to the need for future research clarifying similarities and differences in fear extinction processes across anxiety- and trauma-related disorders [43].

As expected, progesterone levels differed between menstrual cycle groups, with the highest concentrations observed in the mid-luteal phase group, supporting the validity of our cycle phase classifications. Exploratory analyses revealed that higher progesterone levels on the day of exposure were associated with an attenuated improvement in behavioral avoidance, indicating that progesterone may be linked to less favorable exposure therapy outcomes. This may be, at least partly, explained through progesterone’s effects on behavioral avoidance. Specifically, prior findings indicate that progesterone is positively associated with harm-avoidance behavior, and women in the mid-luteal phase (high progesterone levels) have been found to avoid negative stimuli more rapidly than those in the follicular phase (low progesterone levels) [53, 54]. Higher progesterone levels at the time of exposure therapy may, therefore be associated with stronger avoidance tendencies, potentially limiting engagement with feared stimuli and reducing the magnitude of therapeutic improvement.

In contrast, a previous study found that higher progesterone levels during cognitive restructuring predicted greater reductions in behavioral avoidance in women with spider phobia [44]. One possible explanation for these divergent findings may lie in the effects of progesterone’s neuroactive metabolite allopregnanolone (ALLO), which may differ between individuals [28, 55]. In some women ALLO exerts anxiolytic effects by acting as a positive allosteric modulator of GABA-A receptors, whereas others show an increased sensitivity and experience negative mood and anxiety when ALLO is elevated or changing during the luteal phase [56]. Importantly, while this applies to endogenous progesterone in naturally cycling women, synthetic progestins used in HCs exert highly formulation-specific effects on ALLO conversion and receptor binding affinities (e.g., some progestins decrease ALLO levels in the brain, whereas others increase them, or have no effect) [57]. The specific progestin used in HCs may, therefore, differentially shape hormone-related behavioral symptoms in susceptible individuals [58], ultimately contributing to variability in observed effects. Given that increased fear activation has been found to predict poorer post-exposure therapy outcomes [59], inter-individual differences in ALLO sensitivity may modulate fear activation differently, potentially explaining divergent impairing and facilitating effects. Although affective states are thought to cyclically fluctuate across the menstrual cycle, the research remains mixed [48, 60, 61], which may be because some individuals are more sensitive to ovarian hormone fluctuations than others [28]. Therefore, elevated progesterone during the mid-luteal phase, potentially via ALLO, may be associated with heterogeneous affective responses across individuals. The exploratory progesterone median split group effect on behavioral avoidance further emerged only after adjusting for positive affect on the day of exposure therapy, suggesting that heterogeneity in affective responses may have obscured the detectability of progesterone-related group differences in the unadjusted analysis. However, because positive affect was assessed only once, on the day of exposure therapy, it remains unclear whether this heterogeneity reflected menstrual cycle-related affective variation (within-person variability) or stable differences between individuals (between-person variability). Notably, positive affect groups on the day of exposure therapy did not differ in their magnitude of improvement, contrasting previous findings [47]. However, participants with higher positive affect scores showed overall lower phobic fear compared to those with lower scores, though this difference was attenuated after accounting for phobic fear severity. This pattern is more consistent with positive affect reflecting a stable, severity-related individual difference than a cycle-dependent fluctuation, though this should be interpreted cautiously given the single-timepoint assessment of affect. Examining affect as a potentially meaningful phase-dependent variable that interacts with ovarian hormone status in shaping exposure therapy outcomes may thus be particularly relevant for future studies in individuals with affective sensitivity to ovarian hormone fluctuations. Importantly, future studies should account for menstrual cycle-related variability in affect both between and within individuals as such variability may introduce additional heterogeneity and attenuate the detectability of cycle phase effects. In this context, future work may also benefit from considering phase-specific factors that could affect treatment response, such as dysmenorrhea during the early follicular phase, which is associated with concurrent mood [62], or premenstrual symptoms during the luteal phase.

Furthermore, progesterone may also exert its effects through interactions with estradiol [27]. Exploratory analyses revealed that a higher endogenous progesterone-to-estradiol ratio on the day of exposure therapy was associated with reduced behavioral avoidance improvement from pretreatment to posttreatment, indicating that relatively higher endogenous progesterone and lower estradiol levels during exposure-based learning may impair exposure therapy success. Although no menstrual cycle group effects were observed, these results suggest that hormonal states characterized by higher endogenous progesterone than estradiol, such as those typically observed during the mid-luteal phase, may be less favorable for exposure therapy outcomes. A similar discrepancy has also been observed in a previous study. Although menstrual cycle groups did not differ in treatment improvements following exposure therapy, median-split groups based on endogenous estradiol levels did [30]. However, it is important to note that in that study, menstrual cycle phase was self-reported and treatment was not scheduled according to cycle phase, which may have reduced the precision of cycle-phase classification [52]. The discrepancy between menstrual cycle group and hormonal group effects may emerge from inter-individual variability in ovarian hormone levels within cycle phases [63]. Thus, grouping individuals by cycle phase may obscure meaningful hormonal variability that is better captured by direct hormone measurements. In this context, however, assay-related measurement error should also be considered as it may introduce variability unrelated to true hormonal state, particularly in luminescence immunoassays [64]. Nonetheless, these findings highlight the importance of considering the combined effects of ovarian hormones, rather than examining each hormone in isolation.

While it was expected that HC use would be associated with impaired improvement [30, 32], the HC group did not significantly differ in behavioral avoidance and phobic fear improvement from naturally cycling individuals. This impairment has previously been attributed to underlying lower endogenous estradiol levels observed in women using HCs [30]. Indeed, higher endogenous estradiol has consistently been linked to improved extinction learning [21]. In the present study, however, endogenous estradiol on the day of exposure was not associated with improvement in exposure therapy outcomes. Nevertheless, individuals with higher endogenous estradiol on the day of exposure therapy showed overall greater behavioral avoidance and phobic fear compared to those with lower estradiol levels. Contrary to expectation, endogenous estradiol levels did not differ between menstrual cycle groups, and surprisingly, HC users even exhibited the highest endogenous estradiol levels with comparable levels across different HC types. Therefore, the elevated endogenous estradiol levels in the HC group may partly account for the absence of impairment in exposure outcomes expected under HC use. This may explain why higher, rather than lower, endogenous estradiol levels were associated with greater behavioral avoidance and phobic fear as this may reflect effects driven by the HC group, who showed the highest endogenous estradiol levels, irrespective of HC type. Consistent with this interpretation, the HC group also showed a trend for overall greater behavioral avoidance compared to the early to mid-follicular phase group. However, because HC status and endogenous estradiol levels were closely intertwined in this sample, this association cannot be clearly distinguished from a general effect of HC use itself. Moreover, when interpreting findings related to HCs and estradiol, it is important to take some methodological considerations into account. Although estradiol has been theoretically linked to fear extinction outcomes [21], substantial estradiol variability within groups may have reduced the ability to detect between-group differences [63]. This may have been particularly important for the HC group, which encompassed individuals using different types of HCs with varying formulations and endocrine impact [65]. Notably, although endogenous estradiol levels were rather comparable between individuals using oral contraceptives and those using other HCs such as hormonal IUDs containing LNG in the present study, individuals using LNG-IUDs showed descriptively higher endogenous progesterone levels. This aligns, at least partly, with evidence that oral contraceptives suppress endogenous secretion of estradiol and progesterone, whereas LNG-IUDs act primarily through local endometrial effects and preserve ovulation in the majority of cases, independent of cycle regularity [66, 67]. Nevertheless, even in ovulatory cycles, individuals using LNG-IUDs have been found to show lower peak progesterone and preovulatory estradiol levels compared to naturally cycling individuals not using HCs [68]. Hormonal variability may even be substantial within the same HC category. For oral contraceptives for instance, timing of hormone assessment relative to pill intake, or variations in cycle regimen such as active versus inactive pill intake may further contribute to variability, particularly for endogenous estradiol [69, 70]. Larger, more differentiated HC samples are, therefore, needed in future research. Moreover, in contrast to previous studies, we assessed salivary rather than serum ovarian hormone levels [26, 30, 44]. Although feasible and non-invasive, salivary ovarian hormones are less strongly associated with the menstrual cycle than serological assessment [71]. Potential underlying reasons include low hormone concentrations and limited sensitivity of luminescence immunoassays, which is particularly pronounced for estradiol compared to progesterone [64]. Therefore, findings should be interpreted with caution and replication using gold-standard methods such as liquid chromatography-tandem mass spectrometry or serum ovarian hormone assessments are needed. However, in regard to exposure therapy in clinical samples, previous studies also failed to observe an association with endogenous estradiol [44, 72], indicating that estradiol-related effects observed in healthy individuals may not generalize to clinical samples. This discrepancy may again be linked to the transdiagnostic alterations in fear-extinction circuitry described above, which may potentially attenuate or disrupt hormone-related effects in clinical samples relative to healthy controls. However, this interpretation warrants further investigation.

While ovarian hormones were associated with the magnitude of improvement in behavioral avoidance, this was not the case for self-reported phobic fear. This pattern aligns with prior findings suggesting that ovarian hormones may not exert uniform effects across all indices of fear [30, 44, 73]. One possible interpretation is that progesterone may affect the consolidation of inhibitory learning, thereby affecting subsequent behavioral responding when individuals are again confronted with the feared stimulus [44]. Estradiol may be more strongly involved in the short-term expression of extinction learning, including physiological responses such as skin conductance when confronted with the feared stimuli [46]. Self-reported fear, on the other hand, may reflect partially distinct and potentially more stable cognitive-evaluative processes. The timing of outcome assessment may also be critical. In the present study, findings were more consistent for changes from pretreatment to posttreatment assessment than from pretreatment to follow-up assessment, suggesting that hormone-related differences may be more apparent shortly after treatment but may converge over longer follow-up intervals. However, this interpretation remains speculative as previous studies have also observed follow-up effects of ovarian hormones [30, 32]. Studies investigating multiple response modalities across different time points are, therefore, needed to investigate the effects of ovarian hormones during extinction learning and exposure therapy with different short- and long-term outcomes (e.g., avoidance, self-reported fear, and skin conductance), to clarify when and how ovarian hormones may affect exposure-based treatment outcomes.

Previous findings indicated that testosterone may enhance exposure therapy outcomes in individuals with social anxiety disorder [74]. While exploratory analyses revealed no significant association between testosterone on the day of exposure therapy and behavioral avoidance or phobic fear improvement, a trend toward poorer improvement in phobic fear from pretreatment to follow-up was observed among women with low compared to high testosterone levels. The direction of the effects was consistent with prior findings in social anxiety disorder, suggesting a potentially shared, transdiagnostic role of testosterone in supporting exposure-based treatment outcomes. However, beyond limited statistical power, the smaller observed effects may also be partly explained by differing disorder-specific mechanisms as social anxiety involves social-evaluative processes that differ from those underlying specific phobias [75]. Therefore, further research investigating translational versus disorder-specific mechanisms of different hormones involved in exposure therapy outcomes are warranted.

Although previous research indicates that diurnal variation in ovarian hormones is generally small [64] and exposure therapy does not trigger cortisol increase [73, 76], interactions between stress, cortisol, and sex hormone status may nonetheless shape fear learning and extinction processes. For instance, previous studies suggest that the effects of stress and cortisol on fear regulation may depend on sex hormone status in both naturally cycling individuals and HC users; however, findings remain limited and mixed [19, 22]. Additionally, time-of-day effects and associated diurnal variations of cortisol may also play a role [73]. However, neither cortisol during exposure therapy nor time-of-day of the exposure therapy was associated with changes in behavioral avoidance or phobic fear in the present sample, which will be reported in a separate manuscript in more detail. Further research is warranted to clarify how stress and cortisol interact with menstrual cycle phase and ovarian hormone levels during exposure-based learning.

To the best of our knowledge, this is the first study to examine the effects of standardized, biologically informed menstrual cycle phase on exposure therapy success. Menstrual cycle phases were assessed and defined according to established best practice recommendations [45]. Moreover, stringent inclusion and exclusion criteria likely enhanced internal validity by reducing heterogeneity and limiting potential confounding influences. However, several limitations need to be considered, including the small sample size and thus the limited statistical power. Importantly, the COVID-19 pandemic caused a temporary interruption in recruitment and resulted in a smaller sample than originally planned. As a consequence, the study was sensitive to larger effects, and smaller effects may have gone undetected. Null findings should be interpreted cautiously and not as definitive evidence of absence, but as warranting further investigation. For this reason, findings are referred to as preliminary. The relatively homogeneous sample, further limits the generalizability to other populations. Moreover, although consistent with previous investigations [30, 44], menstrual cycle phase and ovarian hormone levels were determined for the day of exposure therapy but not at the pretreatment, posttreatment, or follow-up assessments. Given that anxiety has been observed to fluctuate across the menstrual cycle relative to hormonal changes, particularly among clinical samples [77], cycle phase differences at these assessment time points may have affected behavioral avoidance and self-reported fear on the given day. Consequently, changes in avoidance behavior from pretreatment to posttreatment may reflect not only the effects of exposure therapy but also shifts in underlying menstrual cycle phases and hormonal states across assessment time points. Future studies should assess menstrual cycle phase and ovarian hormone levels at every assessment time point to clarify whether treatment outcomes are more strongly associated with cycle phase and hormonal status on the day of exposure itself or to cycle phase and hormonal status at the time each outcome is measured. Moreover, while endogenous ovarian hormones were assessed, the synthetic hormones delivered through HCs were not measured, resulting in an incomplete hormonal profile for participants using HCs that is particularly relevant for the hormonal median split analyses. Future studies should assess these synthetic hormones to investigate their association with treatment outcomes. A further limitation to note concerns the above-mentioned limited sensitivity of immunoassays for low salivary concentrations. However, this validity concern is less pronounced for progesterone than estradiol [64], and progesterone levels differed between groups as expected, which supports the validity of this result. Moreover, because limited assay sensitivity in this case affects the predictor, random measurement error would be expected to attenuate the association toward null rather than inflate it [78]. Nevertheless, replication using gold-standard methods is highly recommended, particularly regarding the estradiol-related null-findings. Finally, although menstrual cycle groups did not differ significantly on the baseline variables assessed, non-random assignment of menstrual cycle group on the day of exposure therapy may have introduced residual confounding through unmeasured characteristics associated with both cycle phase and treatment response, which future studies may address by scheduling exposure sessions prospectively based on cycle phase.

## Conclusion

The present study provides preliminary evidence that ovarian hormones related to the menstrual cycle on the day of exposure therapy may be associated with changes in behavioral avoidance following exposure therapy in women with spider phobia. The findings, therefore, have the potential to extend previous knowledge about for whom and why psychotherapy works, by identifying when it works [14]. Given that exposure therapy is a broadly applicable treatment approach that is not tailored to sex or gender [79], menstrual cycle phase-informed exposure therapy may be a promising future avenue to improve outcomes among menstruating individuals, if replicated in larger samples [34]. Overall, the findings highlight the potential importance of considering hormonal rhythms in exposure-based interventions, underscoring the need for future studies to confirm and expand the observed findings.

## Acknowledgments

The authors would like to thank all therapists and biologists involved in the implementation of the exposure therapy and biological assessments, particularly Jana Spranger, Leandra Pörtner, Hannah Süss, Jelena Dukic, Corina Passini, Florence Haas, Claudia Casado, and Julia Schmid. We are sincerely grateful to all patients who participated in this study for their time, trust, and commitment. The authors further thank May Ly for careful proofreading of the manuscript.

## Statement of Ethics

This study was performed in accordance with the Declaration of Helsinki. This study protocol was approved by the Ethics Committee of the Kanton Zurich (KEK), Approval No. 2021-00091. All participants provided written informed consent to participate in this study.

## Conflict of Interest Statement

The authors have no conflicts of interest to declare.

## Funding Sources

This study was funded by the Schweizerische Gesellschaft für kognitive Verhaltenstherapie (SGVT). The funder had no role in the design, data collection, data analysis, and reporting of this study.

## Author Contributions

G.Z.: conceptualization, project administration, formal analysis, methodology, writing – original draft, writing – review and editing. P.R.: data curation, writing – review and editing. G.P.: conceptualization, methodology, writing – review and editing. U.G.: conceptualization, methodology, writing – review and editing. U.E.: conceptualization, methodology, writing – review and editing. S.F.: conceptualization, project administration, methodology, writing – review and editing.

## Data Availability

All data generated or analyzed during this study are included in this article and its supplementary material files. Further enquiries can be directed to the corresponding author.
